# Theory-Based Roadmap for Assessing Sustainability in the Collaborative Economy

**DOI:** 10.3389/fpsyg.2021.752867

**Published:** 2021-10-06

**Authors:** Shouheng Sun, Myriam Ertz

**Affiliations:** Laboratory of Research on New Forms of Consumption (LaboNFC), Department of Economics and Administrative Sciences, University of Quebec at Chicoutimi, Saguenay, QC, Canada

**Keywords:** collaborative economy, sharing economy, sustainability, sustainable development, triple bottom line, economy, environment, society

## Abstract

This study aims to investigate the current state of sustainability for the collaborative economy (CE). By utilizing the triple bottom line as a founding conceptual framework, the study summarizes and discusses the sustainability of the CE from three dimensions: environment, economy, and society. The study further proposes some targeted measures and suggestions to measure the level of sustainability of the CE and CE platforms. The result shows that the CE has partially fulfilled some of its initial promises pertaining to sustainability, such as creating new job opportunities, economic growth, the efficient use of space and physical resources, as well as social mixing. However, the current sustainability benefits remain much smaller than some claim and hope for. Therefore, governments, platforms, and the public should work together to solve current challenges pertaining to the CE to tap its sustainability potential.

## Introduction

The redistribution and mutualization of goods and services among peers and various organizations have strongly affected the contemporary economic environment. The collaborative economy (hereafter, CE) refers to “the set of resource circulation schemes that enable consumers to both receive and provide, temporarily or permanently, valuable resources or services through direct interaction with other consumers or through an intermediary” (Ertz et al., 2019, p. 6). Past research has well emphasized that in contrast to traditional markets, where consumers exchange money to gain ownership of new products or access professional services, the CE provides value to consumers by enabling them to access temporarily or permanently a wider set of resources, including pre-owned products, and informally peer-provided services (Belk, 2014). For the past decade, many authors have emphasized that the CE supposedly displays various sustainability advantages. From an economic viewpoint, the CE has often been praised for creating utility between a resource owner and a party in need of that resource at the right time and against reasonable transaction costs. In fact, through drastically different ways of creating, capturing, and disseminating value, the CE incurs multiple utilitarian benefits such as flexible resource provision roles (e.g., online product reseller, car journey provider, money-lender, housing provider), bottom-up self-regulating mechanisms, more authentic experiences for consumers, lower costs, and therefore more sustainable uses of resources (Heinrichs, 2013; Daunorienë et al., 2015). Due to this superior efficiency, the CE has been lauded for being a pathway to environmental and social sustainability (Botsman and Rogers, 2010; Heinrichs, 2013).

The assumptions that the CE can change global and local economies toward greater sustainability (Cohen and Kietzmann, 2014) have been supported by some empirical works using sustainability circles (e.g., Daunorienë et al., 2015). In addition, other influential empirical studies on sharing practices such as toy libraries (Ozanne and Ballantine, 2010), online peer-to-peer swapping (Philip et al., 2019), peer-to-peer renting (Philip et al., 2015), or non-monetary-based private and public sharing events (e.g., Really Really Free Markets [RRFMs]) (Albinsson and Perera, 2012), have further contributed to emphasize the positive impacts that collaborative practices could have on the economy and people, in terms of community and citizenship building. Besides, focusing on the implications of contrived surplus for stakeholders in bike-sharing, home-sharing, and ridesharing, Griffiths et al.’s (2019) conceptual study provided valuable guidelines for developing these sectors sustainably. Unfortunately, this much-needed strand of research on the “sustainabilization” of the CE has been matched with an equivalent if not a more important body of research criticizing the actual contribution of the CE to reach sustainability.

Most of these studies looking deeper into the sustainability nexus of the CE found somewhat mixed results. To Nica and Potcovaru (2015), social sustainability is relative, and the CE has mixed effects on ecological welfare and social connection. Similarly, Joyner Armstrong and Park’s (2017) study of apparel CE found that current apparel CE platforms do not appear to support sustainable consumption, hence sustainability. However, some aspects within specific platforms could undoubtedly be utilized to enhance sustainability factors. To Wu and Zhi (2016), both positive and negative impacts on social, economic, and environmental sustainability exist, and effective design for regulation is needed. The sustainability of the CE can also be questioned by the fact that it induces indulgent consumption or rebound effects (Leismann et al., 2013). In sum, there would be some critical success factors behind the sustainability of the CE. Other authors and observers took a more radical stance toward the CE by asserting that the CE has followed a pathway of corporate co-option that appears unlikely to drive a transition to sustainability (Martin, 2015) and hence granting credit to the early claim that the CE is “neoliberalism under steroids” (Morozov, 2013). A predatory system, better characterized by the “what’s yours is mine” (Slee, 2017) mantra than the earlier “what’s mine is yours” slogan (Botsman and Rogers, 2010).

As Frenken and Schor (2017) predicted, the exact impacts of sharing economy platforms may not be clear for a long time. In sum, tremendous research efforts are still warranted to assess the true contribution of the CE to sustainability. We argue that this lack of contribution is due to two major pitfalls and thus show how we contribute to the literature by rectifying these shortcomings. First, many scholars investigated CE for sustainability in a truncated way by focusing on one or two sustainability circle(s) instead of three. Thus, with a few exceptions (e.g., Daunorienë et al., 2015; Wu and Zhi, 2016), there is a lack of a comprehensive analysis and research on the impact of the CE on the three circles of sustainability. This paper adopts the conceptual framework of the triple bottom line (TBL) to overcome this issue. Second, most writings remain critical but descriptive, providing little prescriptive or normative arguments for alternative ways to develop the CE. Therefore, this paper proposes constructively some targeted measures and suggestions in order to promote the sustainable development of the CE. Third, on a minor point, many studies use a definition of the CE that is biased toward technology-enabled collaborative systems, mainly collaborative platforms or apps which are inherently more prone to criticism due to their lack of physicality and the ensuing issues of tax evasion, user privacy, and employment-related regulation issues that are commonly ascribed to conventional e-businesses (Martin, 2015). Yet, the CE comprises both online and offline resource circulation schemes (Botsman and Rogers, 2010; Ertz et al., 2019). Consequently, we use this broader and more accurate definition that includes both online and offline resource circulation systems, thus watering down the issues related to IT-powered exchange schemes.

## Conceptual Framework

**FIGURE 1:**
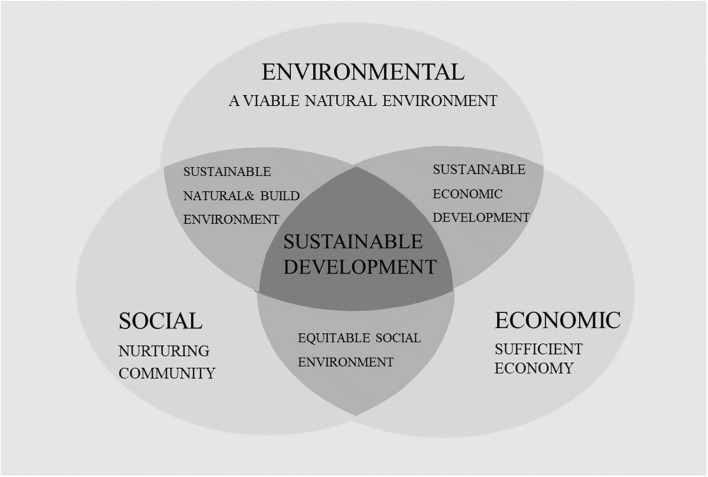
The framework of the triple bottom line.

The concept of sustainability revolves around the idea of the triple bottom line – the social, environmental, and economic components of sustainable practices (Elkington, 1997). This framework originally aimed at measuring corporate performance, considering the traditional economic bottom line and less quantifiable indicators that measure social and environmental impact, i.e., the social bottom line and the environmental bottom line (Ozanne et al., 2016; see Figure 1).

Most importantly, this framework presents two general advantages for methodological accuracy in assessing the contribution of the CE to sustainability. First, it highlights the relationships among these three main components of sustainability (Elkington, 1997, 1998). Ideally, we would like to operate at the intersection of this Venn diagram, where all three goals are satisfied, not only one or two of them, because this lack of comprehensiveness is precisely what got business into the sustainability troubles that it is in today (Figge et al., 2002). In principle, optimal results for any specific program and policy for business, society, and nature are achieved through a triple-win confluence of synergies (Ertz, 2021). Second, each dimension (i.e., circle) can be measured by specific and measurable reference points (Slaper and Hall, 2011). In fact, the three dimensions of sustainability can be measured using indicators that may vary across countries or industries but sharing the commonality of quantitative reporting and assessment that is critical for managerial decision-making.

The TBL sustainability framework has been widely adopted in government, for-profit, and non-profit sectors to evaluate sustainability performance (Aguiñaga et al., 2017; Rezapouraghdam et al., 2019; Song and Moon, 2019; Atisa et al., 2021; Ghannadpour et al., 2021). However, there is no universal standard method for calculating the TBL. This might also be a weakness due to the overall lack of benchmarking and comparison capabilities. However, this lack of standardization can also be viewed as a strength because it allows a user to flexibly adapt the general framework by selecting and adjusting different indicators and evaluation methods for each line according to the specific issues and actual operating conditions (Slaper and Hall, 2011).

In sum, the TBL framework is well-suited for the purpose of this research since it emphasizes both the tripartite and measurable aspects of the sustainability concept. The economic line refers to the impact of the organization’s business practices on the economic system (Elkington, 1997). Economic lines link the organization’s growth to economic growth and how it contributes to supporting the economy. The social line of the TBL framework refers to “the beneficial and fair business practices for labor, human capital, and communities” (Slaper and Hall, 2011). Finally, the environmental line of TBL refers to the efficient use of energy recourses, reducing environmental pollution and greenhouse gas emissions, or minimizing the ecological footprint (Slaper and Hall, 2011).

## Methodology

The systematic literature review is a widely used method that synthesizes a large number of studies and provides insights into current advances and remaining research gaps. In order to summarize the initial sustainability assumption and investigate the current state of sustainability for the collaborative economy, this study follows a systematic literature review of the Campbell/Cochrane type (Bearman et al., 2012), which includes nine steps: Elaboration of research questions, determination of inclusion and exclusion criteria, elaboration of research nomenclature, independent analysis and coding, mapping of the results in an analysis grid, independent summary of publications by coders, publication summary writing. The detailed processes of the systematic literature review are shown in Table 1.

**TABLE 1:** The processes of systematic literature review.

No.	Stage	Process description
1	Elaboration of research questions	What are the initial sustainability assumptions about the collaborative economy? What is the current state of sustainability for the collaborative economy?
2	Determination of inclusion and exclusion criteria	The study encompasses documents published as of February 2021. The search considered peer-reviewed articles, conference proceedings, book chapters, professional articles, industry statistics, and research reports. Besides, only papers published in English were selected.
3	Elaboration of research nomenclature	A series of systematic retrievals were performed in major academic databases: ABI/Inform, Academic Search Complete, Google Scholar, JSTOR, Scopus, PubMed, Web of Science, and IEEE Xplore. Search terms were selected as follows: “sharing economy” or “collaborative economy” or “collaborative consumption” or “community-based economy” or “access economy” or “rental economy” or “on-demand economy” or “mesh economy” or “car sharing” or “ride sourcing” or “ride sharing” or “ride hailing” or “mobility sharing” or “shared mobility” or “uberization” or “uberization” or “mutualized mobility” or “vehicle sharing” or “bike sharing” or “bicycle sharing” or “E-Scooter sharing” or “platform economy” or “gig economy” or “p2p economy” or “peer to peer economy” or “peer economy” or “peer-to-peer economy” or “peer2peer economy” or “peer 2 peer economy” or “peer-2-peer economy” or “reselling and trading” or “secondary market” or “second-hand market” or “second-hand economy” or “talent sharing” or “niche services” or “diy economy” or “crowd economy” or “crowdsourcing” or “crowdfunding” or “collaborative fashion consumption” or “food sharing” “meal sharing” or “clothes sharing,” “tool sharing,” “space sharing” or “accommodation sharing” or “coworking” or “office sharing,” “home sharing” or “goods sharing”. The search returned 3,051 documents that contain any of these terms within the title, abstract, or keywords of the original works. After removing 136 duplicates, 2,915 publications were analyzed thoroughly by examining all parts of the text.
4	Independent analysis and coding	All selected documents were then submitted to two analysis and sorting rounds. Two independent coders analyzed those documents during each round according to the relevance to the research question and the inclusion and exclusion criteria. After discussing and exchanging ideas, the process ended with selecting 239 documents considered for the systematic review.
5	Mapping of the results in an analysis grid	Based on the conceptual framework of this study, that is, the TBL sustainability framework, the research results of the documents were mapped to assess their relationships. Each document was described in detail in an Excel analysis grid in terms of the full title, author(s), year of publication, publication titles or source titles, primary findings, and dimensions of the TBL sustainability framework. It is worth noting that a document may correspond to more than one dimension of the TBL sustainability framework.
6	Independent summary of publications by coders	The two coders then independently wrote a summary of each document emphasizing the objectives, results, and critical contributions. Finally, both met to exchange under the direction of a supervisor to confront their work and identify converging themes.
7	Publication quality and rigor assessment	The publication quality and rigor assessment were conducted by several means, such as the source of the document, the soundness of the methods used, the logical anchoring of the results, and the contributions of findings.
8	Publication summary writing	A content analysis includes writing the highlights containing the objectives, methods, results and findings, and contribution of each publication. The condensed versions of these summaries were developed in the Excel matrix, emphasizing the dimensions of the TBL sustainability framework involved in each publication. This step provides a preliminary division of sustainability dimensions for each article based on the TBL framework, namely economic, environmental and social.
9	Theme identification	According to the content analysis and dimension division in step 8, we further identified the themes under each dimension of TBL framework (i.e., economic, environmental and social). For example, in economic dimension, 5 themes of “Increase personal income and welfare,” “Creating new job opportunities,” “Revitalizing the traditional industry,” “Improving the distribution of wealth and resources,” and “Promote business prosperity and economic growth” were identified. In this step, text summaries and Excel grids obtained in step 8 were further refined according to the dimensions and themes. Based on this, we summarized the originally assumed sustainability prospects of the CE and investigate the current state of the sustainability of the CE.

## Results

### The Originally Assumed Sustainability Prospects of the Collaborative Economy Using the Triple Bottom Line

The early enthusiasm for the CE was driven mainly by its anticipated and overtly hypothetical sustainability impacts (Benkler, 2004; Botsman and Rogers, 2010). As a new economic model, it was believed that the CE could bring a fresh new vitality to the traditional industry market and stimulate economic activity. As a result, it was assumed to be a unique economic growth point for countries or regions, ushering in a new era of novel employment opportunities and lower unemployment rates (Watanabe et al., 2016). Another positive impact of the CE is the rise in income or consumer welfare. On the one hand, the lower transaction costs facilitate the transactions between strangers and thus provide more opportunities for providers to use idle resources for additional income. On the other hand, at the same time, lower prices and more choices of consumption can make consumers obtain more benefits. In addition, it is also believed that low-income groups and disadvantaged communities are more likely to benefit from the CE, thereby improving the distribution of wealth and income (Brachya and Collins, 2016).

The environmental promise of collaborative modes of exchange held that it would enable consumers to reduce dependence on private ownership (Frenken, 2017). Instead, with cheap and easy access to goods owned by other individuals or organizations, they would gradually detach themselves from ownership and favor access-based consumption (Belk, 2014). In doing so, consumers would save money and contribute to lower material demand and energy use. In addition, the total amount of new products needed by the entire society can also be reduced. Therefore, the CE is considered a promising way to help facilitate the sustainability transition. A typical example is a car-sharing program, which claims to reduce the total number of cars and the required mileage, thereby helping to reduce air pollution and greenhouse gas emissions. In addition, another benefit comes from the improvement of space utilization efficiency. For example, fewer vacant parking spaces and buildings allow for a higher density of urban living and increase related energy efficiency per capita (Bettencourt et al., 2007). More importantly, sharing can provide a flexible infrastructure system that can more effectively deal with peak demands in emergencies or mega-events with fewer public investments, such as earthquakes, hurricanes, World Cups, Olympic Games, etc. (Frenken, 2017).

Apart from the economic and environmental benefits, there are also some claimed social benefits. With new technologies, particularly the Internet, sharing propelled existing practices to a much larger social scale. Furthermore, CE practices increase opportunities for contact and communication between people from different social backgrounds. That is, sharing practices promote social mixing (Frenken and Schor, 2017). In addition, it is often mentioned that the CE can help build community networks and strengthen community resilience and social connectivity (Nica and Potcovaru, 2015; Brachya and Collins, 2016), which is especially beneficial in a time of need, such as providing immediate assistance in response to an emergency.

Moreover, the basis of transactions between the provider and consumer in the CE is the “digital trust,” which is established by verifying the authenticity of the providers and consumers as well as online rating and review systems. This mechanism for building trust among strangers will continue to grow and improve with CE development and help develop a more trusting society (Nica and Potcovaru, 2015; Wu and Zhi, 2016). Last but not least, the CE has the potential to supplement incomes while lowering the cost of living. Therefore, it could potentially provide opportunities for those lower-income people with the time and skills to become upwardly mobile, promoting social equity to some extent (Brachya and Collins, 2016).

**FIGURE 2:**
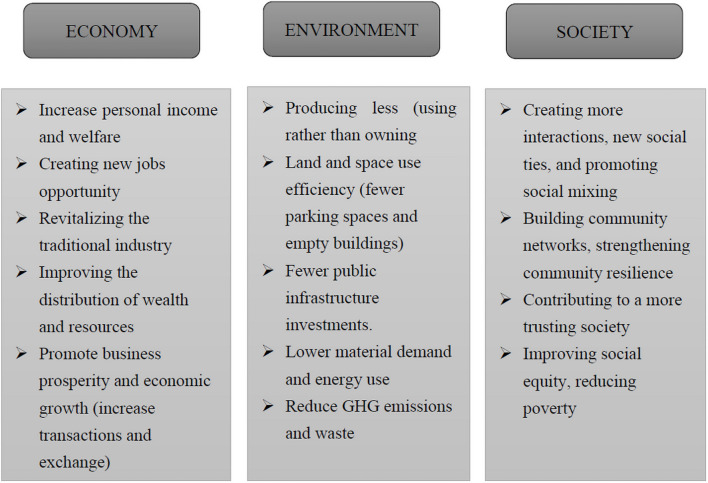
The initially assumed sustainability prospects of the CE.

We draw on a substantial body of literature that recapitulates the major sustainability assumptions pertaining to the CE and summarizes the initially assumed drivers for sustainability related to the CE in Figure 2 and Table 2.

**TABLE 2:** The originally assumed sustainability prospects of the CE.

Assumptions	Some representative studies	Authors and year
***Economy***		
Increase personal income and welfare	Sharing nicely: On shareable goods and the emergence of sharing as a modality of economic production	Benkler (2004)
	What’s mine is yours: The rise of collaborative consumption.	Botsman and Rogers (2010)
	From Zipcar to the sharing economy	Sundararajan (2013)
	You are what you can access: Sharing and collaborative consumption online.	Belk (2014)
Creating new job opportunities	Commercial orientation in grassroots social innovation: Insights from the sharing economy	Martin et al. (2015)
	Impact of shared economy on urban sustainability: From the perspective of social, economic, and environmental sustainability.	Wu and Zhi (2016)
Revitalizing the traditional industry	Ride On! Mobility Business Models for the Sharing Economy	Cohen and Kietzmann (2014)
	How traditional firms must compete in the sharing economy.	Cusumano (2014)
	Evaluating the sustainability of sharing economy business models	Daunorienë et al. (2015)
	The rise of the sharing economy: Estimating the impact of Airbnb on the hotel industry	Zervas et al. (2017)
Improving the distribution of wealth and resources	Sharing economy: a potential new pathway to sustainability.	Heinrichs (2013)
	The sharing economy and sustainability	Brachya and Collins (2016)
	Putting the sharing economy into perspective	Frenken and Schor (2019)
Promote business prosperity and economic growth (increase transactions and exchange)	Sharing nicely: On shareable goods and the emergence of sharing as a modality of economic production	Benkler (2004)
	Operationalization of un-captured GDP-Innovation stream under new global mega-trends	Watanabe et al. (2016)
	The sharing economy and sustainability	Brachya and Collins (2016)
***Environment***		
Producing less (using rather than owning	Collaborative consumption: toward a resource-saving consumption culture	Leismann et al. (2013)
	You are what you can access: Sharing and collaborative consumption online	Belk (2014)
	The sharing economy: make it sustainable	Demailly and Novel (2014)
	Political economies and environmental futures for the sharing economy	Frenken (2017)
Land and space use efficiency (fewer parking spaces and empty buildings)	Growth, innovation, scaling, and the pace of life in cities	Bettencourt et al. (2007)
	Sharing economy: a potential new pathway to sustainability	Heinrichs (2013)
	Adapting to the sharing economy	Matzler et al. (2015)
Fewer public infrastructure investments	Growth, innovation, scaling, and the pace of life in cities	Bettencourt et al. (2007)
	The sharing economy and sustainability	Brachya and Collins (2016)
	Political economies and environmental futures for the sharing economy	Frenken (2017)
	Putting the sharing economy into perspective.	Frenken and Schor (2017)
Lower material demand and energy use	Collaborative consumption: toward a resource-saving consumption culture	Leismann et al. (2013)
	Sharing economy: a potential new pathway to sustainability	Heinrichs (2013)
	The sharing economy: make it sustainable	Demailly and Novel (2014)
	Putting the sharing economy into perspective	Frenken and Schor (2017)
Reduce GHG emissions and waste	Greenhouse gas emission impacts of carsharing in North America.	Martin and Shaheen (2011)
	Sharing economy: a potential new pathway to sustainability	Heinrichs (2013)
	The sharing economy and sustainability	Brachya and Collins (2016)
	Putting the sharing economy into perspective.	Frenken and Schor (2017)
***Society***		
Creating more interactions, new social ties, and promoting social mixing	Alternative marketplaces in the 21st century: Building community through sharing events	Albinsson and Perera (2012)
	Sharing economy: a potential new pathway to sustainability	Heinrichs (2013)
	Putting the sharing economy into perspective.	Frenken and Schor (2017)
Building community networks, strengthening community resilience	Alternative marketplaces in the 21st century: Building community through sharing events	Albinsson and Perera (2012)
	The social sustainability of the sharing economy	Nica and Potcovaru (2015)
	The sharing economy and sustainability	Brachya and Collins (2016)
Contributing to a more trusting society	The social sustainability of the sharing economy	Nica and Potcovaru (2015)
	Impact of shared economy on urban sustainability: From the perspective of social, economic, and environmental sustainability	Wu and Zhi (2016)
Improving social equity, reducing poverty	Sharing economy: a potential new pathway to sustainability	Heinrichs (2013)
	The Promise of the Sharing Economy among Disadvantaged Communities	Dillahunt and Malone (2015)
	The sharing economy and sustainability	Brachya and Collins (2016)
	The sharing economy: Why people participate in collaborative consumption.	Hamari et al. (2016)
		

Despite these early assumptions emphasizing the prospects of the CE in promoting economic, environmental, and social sustainability from different perspectives, after years of development and practice, it became clear that many challenges persist with regard to the CE. Therefore, the following section elaborates on the main points of contention that remain in the CE within each of the three sustainability circles.

### Re-examination of the Sustainability Potential of the Collaborative Economy Using the Triple Bottom Line

With the rapid development of the CE, many adverse effects have appeared, casting a shadow over the prospect of sustainability. Therefore, whether the current CE is achieving the sustainability that was initially assumed remains debatable. Using the TBL framework, we re-examine the sustainability of the CE from economic, environmental, and social viewpoints.

#### Economic Dimension

The direct economic effects of the CE are noticeable and positive. The US Federal Trade Commission (2016) released a report discussing the economic implications of the rise of the CE, which emphasized the significant consumer benefits brought about by the competition between collaborative organizations and traditional industries. In addition, the CE has injected new vitality into the market. A large number of enterprises rose rapidly and have made noticeable achievements. The market value of some of the CE platforms, such as Uber, Airbnb, and DiDi has surpassed long-established firms in the sector.

The CE has also played a positive role in improving employment and economic development. According to the China State Information Center (2019), the transaction volume of China’s CE market in 2018 was approximately 432.65 billion dollars, an increase of 41.6% over the previous year. In Canada, the CE has become an annual $1.3 billion industry that is slightly smaller than fishing, hunting, and trapping (Canada West Foundation, 2019). The size of the CE in the entire European Union (EU) economy was estimated at 26.5 billion euros in 2016, 0.17% of EU-28 GDP (Naumanen et al., 2018). In terms of employment, an estimated 162 million people are providers on sharing platforms in the United States and Europe alone (Manyika et al., 2016). In China, the number of people involved in providing products and services was about 75 million, and the number of platform employees was 5.98 million in 2018 (China State Information Center, 2019). Given that we also consider offline CE schemes, these figures may be much higher.

However, the total economic impact of the CE is much more complicated. The rise of CE has also triggered some adverse effects on the related industries and markets. Traditional sectors are likely to experience lower employment rates and lower earnings due to the cannibalization of collaborative schemes. For example, the income of conventional hotels fell sharply in places where home-sharing grew, especially in the lower-end and cheaper segments of the hostel market (Zervas et al., 2017). Similar effects also exist in the traditional car rental market. With the rise of emerging car-sharing platforms, classic car rental companies are facing increasingly fierce competition. Likewise, ridesharing platforms, most notably Uber, take taxi market shares (Ertz et al., 2018). In addition, home-sharing also has a potential impact on housing supply and prices. In neighborhoods where home-sharing is widespread, the residents would see their rents rise. It has been shown that Airbnb services increase house prices and diminish affordability (Sheppard and Udell, 2016).

Another issue worth noting is that the distribution of increased income and welfare from participating in the CE may be uneven (Frenken, 2017; Stemler, 2017). First, the CE platforms are bilateral platforms with solid network externalities, which tend to form an industry monopoly and grab high margins (Frenken, 2017).

Moreover, the most profitable participants tend to be the well-off people with valuable assets because they can more easily use the assets to earn rents continuously (Schor and Attwood-Charles, 2017). This phenomenon is most apparent in home-sharing and applies to renting out limousines, parking spaces, boats, and high-end fashion brands, in times and places where such goods are scarce (Frenken and Schor, 2017). Thus, in general, although CE participants are experiencing increased consumer welfare brought about by more varieties and lower prices, the economic inequality driven by the power of providers may also increase.

Collaborative economy platforms utilized the funding from venture capitalists to subsidize consumers for years. However, these welfares are also unevenly distributed. These gargantuan funds have been used to subsidize the lifestyles of wealthier city dwellers (Smith, 2016). According to the Internet Use Survey conducted by the U.S. Census Bureau in 2017, CE services in the US are used mainly by wealthy, well-educated urbanites (Yahoo Finance, 2019). It can thus be seen that the CE hasn’t done so well in sharing wealth so far.

Large capital inflows have usually characterized the introduction of some collaborative organizations on financial markets. Therefore, it is easy to lead to excessive concentration of investment in the CE. It is hard to say whether the CE is a carnival or a nightmare for capital markets, but the prospects are not so good since many CE giants such as Uber or WeWork generate very few profits. Simultaneously, to ensure user growth, the platform usually stimulates users at both ends of supply and demand to participate in the platform through financial subsidies (Sun and Ertz, 2021). That is not a profitable, sustainable development model. Therefore, CE platforms have increasingly become worrisome due to their perceived lack of financial sustainability. In particular, some weird and unusual products and services have constantly emerged in the CE market, such as a friend, basketball and small camp stool, chicken for obtaining fresh eggs, goats as lawnmowers, other people’s toilet (Grigoras, 2016), which also pose a significant challenge to the original purpose and profitability of the CE.

Another issue worth noting is the monopoly issue. Driven by economies of scale, the CE can quickly form industry monopolies, such as Uber and DiDi, in the ride-hailing market. This will undoubtedly bring certain pressure and challenge to the healthy competition and development of the whole industry. In addition, as there are fewer competitors, the product and service prices can be set according to the giant’s will, such as the surging price system of Uber. Although the dynamic pricing system has been widely adopted in the online market, its power is well known: the price is set by competing individuals, and the supply of goods is transparent. However, the pricing algorithm of Uber is opaque. Moreover, its reliance on black-box algorithms makes the pricing system more vulnerable to manipulation than other online marketplaces. Thus, this pricing system can harm the interests of consumers and the dynamics of free and open markets to a certain extent.

**FIGURE 3:**
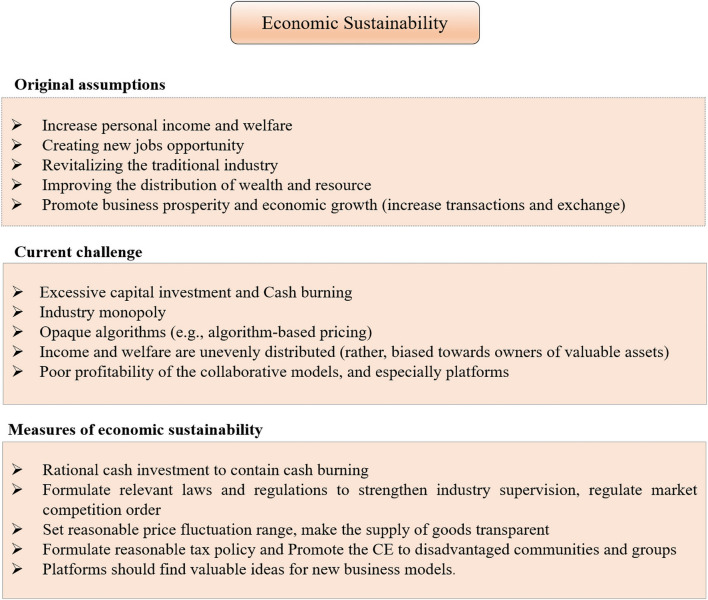
Current challenge and measures for the economic sustainability of the CE.

As mentioned above, we summarize the current challenge for the economic sustainability of the CE in Figure 3.

Here, we propose some suggestions to address these issues in order to better promote the economic sustainability of the CE. First, the government should formulate relevant laws and regulations to strengthen industry supervision and regulate market competition in order to control monopolistic behavior. In addition, a reasonable and effective CE tax system should be established to promote wealth distribution and reduce income inequality. Second, platforms should conduct sound cash investments and find valuable ideas for new business models in order to promote the financial sustainability of the platform. Third, as to the pricing strategy, platforms should set a reasonable price fluctuation range and open up more information about pricing, such as the available supply.

#### Environmental Dimension

The CE can promote sustainable use of resources in some fields, such as renting out an idle room or house, sharing expensive hand tools and garage equipment, or even sharing surplus food and clothes (Stanoevska-Slabeva et al., 2017). All of these can help to improve the use efficiency of space and physical resources and reduce waste. Thus, even though it may directly contribute to environmental sustainability to a small extent, it also provides a way to redistribute and reuse commodities instead of just discarding them.

However, although the CE is considered environmentally friendly, such as by reducing carbon emissions and environmental pollution, there is no reliable empirical evidence to support these claims.

The current research on the environmental impact of the CE mainly focuses on the first round of effects, that is, the direct substitution effect, and often ignores the rebound effect. There are two types of rebound effects, namely direct rebound effects and indirect rebound effects. Direct rebound effect refers to the situation where rebound effects occur within the same resource or service system (Pouri, 2021). For example, affordable sharing services (e.g., car-sharing, ride-hailing, accommodation sharing, talent sharing, and niche services) provided by the platform enable consumers to use more of the platform’s services (Leismann et al., 2013; Harris et al., 2021; Pouri, 2021). Indirect rebound effect refers to the situation where the rebound effect occurs outside the same resource or service system (Meshulam et al., 2021; Pouri, 2021). The expenses saved and the earnings generated by participating in the CE may also stimulate a new round of consumption in other areas or sectors. For example, the money savings from the renting or reselling of old goods, affordable accommodation sharing and coworking services, or the earnings from talent sharing or niche services may be re-spent on other goods and services such as new electronic products, fashionable clothes, and air travel (Harris et al., 2021; Meshulam et al., 2021; Pouri, 2021).

As a result, the environmental benefits from the initial sharing activities are likely to be offset. For example, car-sharing may make car trips more efficient and available, increasing the number of such trips and offsetting environmental gains achieved by curbing individual car usage (Leismann et al., 2013). Therefore, a more in-depth examination is needed to explore changes in user behavior patterns and consumer psychology to accurately assess the impact of the rebound effect to determine whether the potential for resource conservation and environmental protection can be achieved without a significant rebound effect. Reducing or avoiding the negative impact caused by the potential rebound effect is also a significant challenge facing the related sectors and platforms of the CE. Adopting advanced energy and environmental technologies, optimizing resource allocation, continuously improving resource utilization efficiency, and minimizing the resource and environmental impact of the entire product or service sharing cycle is a potential direction for sustainability (Harris et al., 2021).

In addition, many CE platforms are continuously creating new markets and expanding trade volume, which injects more purchasing power into the economy. These new economic activities are more likely to expand the total market demand, increase energy consumption, and put more pressure on the environment.

In short, the impact of the CE on the environment is highly complex, which needs to be scientifically assessed through comprehensive and systematic studies. However, so far, there is still a lack of related research results in this area. One of the main reasons is that CE platforms are unwilling to disclose actual operating data for independent research, given concerns about privacy and trade secrets (Frenken and Schor, 2017). Instead, some CE platforms commissioned their environmental research, which renders the validity of these findings hard to judge.

Here, we combine the development and practice of the CE in recent years to re-examine its environmental impact. First, some projects also caused the excessive supply of products, leading to a severe waste of material resources. In some fields of the CE, platform enterprises often adopt the strategy of constantly providing products and services to the market in order to enhance their market penetration rate and obtain a competitive advantage. The most striking example was free-floating bike-sharing in China. Between 2017 and 2018, more than 20 million bicycles flooded into major cities in China (China State Information Center, 2019), and that oversupply of bicycles has resulted in an extreme strain of urban space resources and entire landfills littered with colored bikes.

Although ridesharing platforms have long claimed to be environmentally friendly, more and more data, indicate that these companies are exacerbating congestion on the roads and undermining the sustainability of urban transportation. According to the San Francisco County Transportation Authority (2018), compared with 2010, traffic congestion in San Francisco increased by about 60% in 2016. More than half of that increase was attributed to Uber and Lyft. In a 2016 survey conducted by the University of California, ridesharing services reduced Americans’ use of bus systems and light rail services by 6 and 3%, respectively (Clewlow and Mishra, 2017). In addition, the statistics of the U.S. Census Bureau show that car ownership has increased over the past 5 years (between 2012 and 2017) in the eight major cities where Uber and Lyft are most concentrated, a worrisome reversal of earlier trends (Schmitt, 2019). In particular, a study released by Uber and Lyft in 2019 admitted that their fleet contributed to congestion and the decline of public transit ridership in six major cities surveyed (Fehr and Peers, 2019).

**FIGURE 4:**
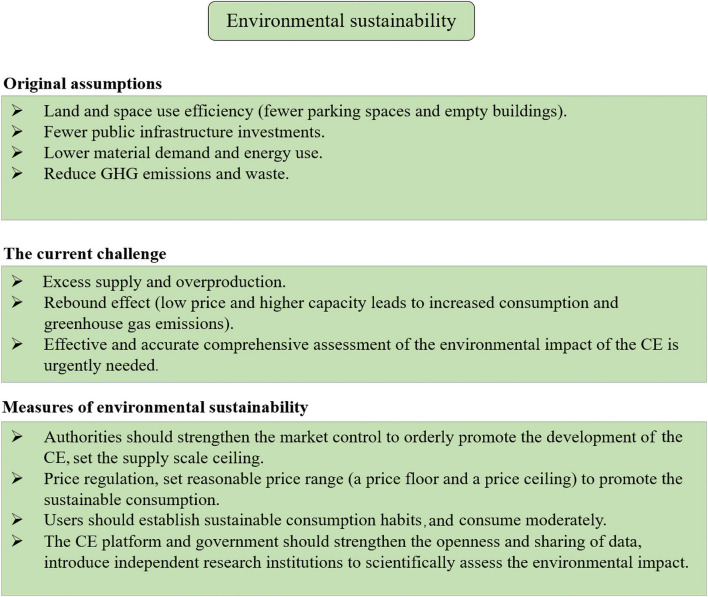
Current challenge and measures for environmental sustainability of the CE.

As mentioned above, we summarize the current challenge for the environmental sustainability of the CE in Figure 4.

Even though the CE has some potential in the transition to environmental sustainability, the transition will not be painless. So here we propose some suggestions to address the current challenge in order to better promote environmental sustainability.

First, authorities should strengthen the market control to orderly promote the development of the CE. For example, they should set the supply scale ceiling to limit the excess supply and overproduction, implementing price regulation to curb too low prices stimulating excessive consumption (i.e., rebound effect). In addition, participants in the CE should also set up sustainable consumption habits and adhere to moderate and rational consumption to prevent excessive consumption and resources waste. Last but not least, an effective and accurate comprehensive assessment of the environmental impact of the CE is urgently needed. Therefore, the CE platform and government should strengthen the openness and sharing of data and introduce independent research institutions to scientifically assess the environmental impact in order to provide valuable reference and guidance for better environmental sustainability.

#### Social Dimension

As to the social dimension, CE is widely believed to bring many benefits in terms of getting to know others and making new friends (Fitzmaurice et al., 2020). However, while the CE certainly instills authenticity and human contact within a transaction, these social benefits are not the norm. For example, only half of Airbnb hosts seem to regard social interaction as the core of their motivations and practices (Frenken and Schor, 2017). Among them, socially oriented hosts are eager to interact with “comfortable exotic” foreign guests (Ladegaard, 2018). In addition, some TaskRabbit (an online marketplace that matches freelance labor with local demand) users said that the platform provides them with opportunities to meet new friends, and they are satisfied with this new type of social network (Fitzmaurice et al., 2020).

Nonetheless, the CE is also confronted with several challenges that may affect its social sustainability. Some pressing issues are discrimination, labor right, and trust problem – first, the peer-to-peer discrimination in the CE. A study on Airbnb in the United States found that Afro-American guests are rejected by hosts more frequently, and the rents of Afro-American male hosts are about 12% lower than those of other hosts for the same type of house in the same kind of location (Edelman et al., 2017). This discrimination has also been found in the ride-hailing industry. Afro-Americans faced longer average waiting times and more frequent cancelations than white passengers (Ge et al., 2016). Second, due to the imperfect laws and regulations of the CE, some CE platforms maximize their business interests by utilizing flexible employment and labor outsourcing and evading government regulation. It does not provide employees with the same social security and welfare as the traditional industry, which has seriously harmed the rights and interests of workers.

Moreover, trust is essential in situations of risk, uncertainty, and interdependence (McKnight and Chervany, 2001). These three elements are very prominent in the CE (Gruber, 2020; Nienaber et al., 2021). The regular operation of the CE platform is based on the trust and collaboration between the user groups at both ends of the supply and demand (Bhappu et al., 2020; Gruber, 2020). Peer reviews and ratings can foster honesty and transparency, which are critical components of a successful CE platform. Sadly, reviews on sharing platforms can be faked (Stemler, 2017). However, even reviews and evaluations made in good faith can be confusing. For example, there is empirical evidence that some platforms encourage the production of positive reviews (Zervas et al., 2017) instead of ones with a critical bent, thus leading to the overproduction of “good” scores. This constitutes a major problem for users as it inflates ratings, essentially reducing their informative value and further lead to several concerns about trust and safety. For example, housing owners may sometimes experience severe damage to their properties, while the driver’s language provocation and physical harassment of the passengers frequently occur (Statt, 2019). Since the CE is not neatly classified into the traditional legal category, it is still in legal gray areas and faces regulatory uncertainty. Therefore, these concerns may lead to a lack of trust in participating in the CE and might erode future transactions (Hawlitschek et al., 2016).

Last but not least, the rapid expansion of the CE has also led to some problems that seriously threaten the urban system and order and bring significant pressure and challenges to urban and community governance (Ma et al., 2018). Therefore, grasping the balance between the cultivation of new business forms and the comprehensive governance of the city, society, and market is a test of the wisdom of government managers.

**FIGURE 5:**
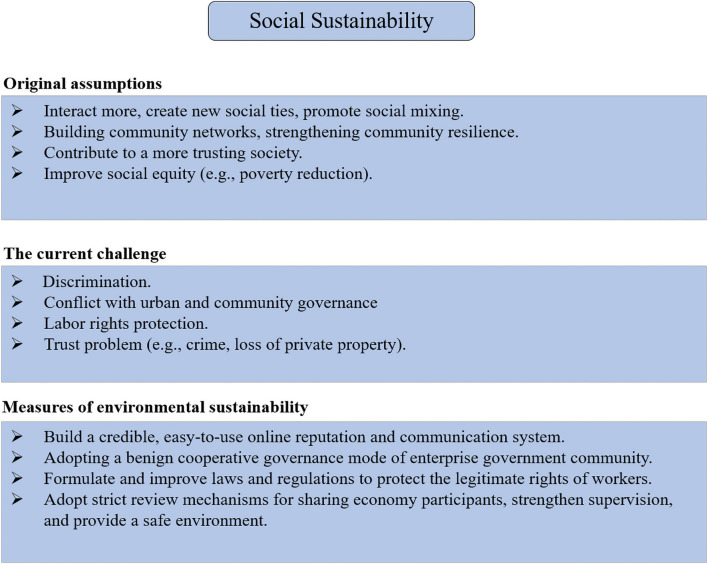
Current challenge and measures for social sustainability of the CE.

As mentioned above, we summarize the current challenge for the social sustainability of the CE in Figure 5.

Here, we propose some suggestions to address the current challenge in order to better promote the social sustainability of the CE. First, as to the conflict with urban and community governance, it is better to adopt a cooperative governance model involving organization, government, and community participation. This can reduce the friction encountered on the way forward of the CE and enable the CE to be better promoted in the community. Second, in terms of labor rights protection, authorities should formulate and improve relevant laws and regulations to protect workers’ legitimate rights and interests as soon as possible.

As for discrimination, according to the empirical study of Cui et al. (2020), enough shared information can reduce discrimination. Especially, the verifiability and credibility of the peer-generated reviews is crucial for reducing discrimination. Therefore, platform companies should encourage users and providers to actively and objectively participate in peer-generated reviews and build a credible, easy-to-use online communication and reputation system. In short, disclosing information rather than hiding information from users is more likely to resolve discrimination in the CE successfully.

In terms of the trust problem, this is mainly caused by the lack of supervision of the CE. On the one hand, the existing laws and regulations are not yet fully applicable to the CE model with its digital-driven and peer-to-peer nature. On the other hand, this can easily lead to the lack of necessary supervision in the transaction process of products and services. For example, hotels are checked to ensure quality, while Airbnb apartments are not.

On the other hand, platforms often encourage positive peer reviews to attract more users and enhance brand impact, which essentially invalidates the entire credit evaluation system of the CE. These have led to the constant emergence of platform trust and security issues. Therefore, platforms should adopt strict review mechanisms for CE participants. Furthermore, authorities should formulate relevant regulations to strengthen CE platforms’ operation supervision to provide a safe transaction environment.

## Discussion

This study organizes existing research on the collaborative economy in order to investigate its current state of sustainability. The two questions that guided the research were: Does the current CE achieve its original intention of sustainable development? And how to improve the sustainability of the CE? This paper draws on the triple bottom line as a conceptual framework to summarize and discuss the sustainability of the current CE from three dimensions: environment, economy, and society, and concludes by proposing some targeted measures and suggestions to promote the sustainable development of the CE.

In sum, it can be seen that the claimed benefits of the CE on sustainability are much more complex than initially expected. Although the CE has good potential for sustainability, the current performance is not satisfactory. The contribution of the CE to the economy is direct and prominent. It has created new job opportunities and increased people’s welfare, contributing to business prosperity and economic growth. However, some challenges affect economic sustainability, such as excessive capital investment and cash burning, industry monopoly, opaque algorithm-based pricing, income, uneven distribution of welfare, and financial profitability of the CE platform. In addition, the performance of the CE in environmental sustainability is far from meeting the original expectations, which is most prominent in the field of mobility-sharing services. Some major problems such as excess supply and overproduction, rebound effect (mainly refers to the excessive consumption), and lack of accurate, comprehensive assessment of the environmental impact of the CE need to be urgently solved. In terms of social sustainability, although it has indeed promoted social mixing to some extent, the social problems brought by the current CE, such as peer-to-peer discrimination, labor rights, trust and safety problems, seem to be more than the beenfits it initially assumed.

Therefore, it is essential to consider effective strategies to mitigate the adverse effects and promote the sustainability of the CE. Governments, platforms, and the public need to work together to address the sustainability challenges of the current CE and better promote the transition to sustainability.

The government should formulate laws and regulations to strengthen industry supervision to control platforms’ operations such as monopolistic and pricing behavior, transaction security mechanism, and the supply scale ceiling; it also needs to establish a reasonable and effective tax system and labor rights protection law for the CE.

Instead of cash burning, the platform should conduct sound cash investment and find valuable ideas for new business models to promote financial sustainability. Furthermore, the platform should fully bear its social responsibility and consider the impact of its economic behavior on society and the environment. For example, limiting the excess supply and overproduction, curbing too low prices stimulating excessive consumption, providing a more transparent and reasonable price fluctuation range, building a credible reputation and communication system, improve working conditions and welfare for laborers, among others.

It is worth noting that consumer criteria also affect the sustainability of the CE to a certain extent. The relative importance of consumers’ economic, social and environmental motivations to participate in CE vary significantly among social demographic groups, users and providers, and different types of shared goods and services (Hamari et al., 2016; Böcker and Meelen, 2017; Parguel et al., 2017). For example, the consumers’ motivations to participate in expensive accommodation sharing services may be highly economical, while environmental motivations may be important for the users of mobility sharing. In terms of CE forms with high levels of interpersonal communication, such as meal sharing or co-working, social motivation plays a critical stimulating role (Hamari et al., 2016; Böcker and Meelen, 2017; Parguel et al., 2017). However, the actual sustainability impact remains mixed. Driven by different consumption motivations, the consumption behavior of users in CE may promote environmental sustainability by reducing the demand for new products and their corresponding production while improving the effective use of existing products. However, as mentioned before, collaborative behaviors may also lead to negative rebound effects whether directly (i.e., increase in product or service usage intensity) (Leismann et al., 2013; Harris et al., 2021; Pouri, 2021) or indirectly (i.e., shifting the gains and savings obtained from the CE to other potentially less sustainable consumption activities) (Meshulam et al., 2021; Pouri, 2021). Consumers’ initial intentions and motivations are therefore crucial in this regard since those primarily motivated by sustainable objectives will also seek to curb adverse rebound effects, while consumers motivated by other criteria such as lower prices/financial gains, better commodity or social prestige, might be less conscious about adverse effects (e.g., rebound effects) or less prone to act in line with sustainable motives.

Considering that the consumer’s motivation can be changed (e.g., from economic motivations to environmental concerns), how to better understand users’ consumption behaviors and identify deeper consumers’ motivations, and actively guide CE users’ motivations and behaviors to transform to sustainable consumption patterns, is a promising way to improve the sustainability of the CE. Moreover, the CE emphasizes the scale effect and network effect at both ends of supply and demand. Empirical evidence suggests that individuals with prior usage experience showed higher future usage intentions for the sharing service (Hamari et al., 2016; Böcker and Meelen, 2017). Ertz et al. (2021) conceptually introduced and empirically substantiated the switchover effect in CE (i.e., individuals switch from being a user to a provider). They found that personal values, learning experience, social benefits, mutuality, and peer influence can drive users to become providers. While distrusting strangers, a sense of intimacy, a lack of resources to share, and a lack of skills inhibit the switchover process. Therefore, strengthening the driving factors and reducing (or eliminating) the obstacles to increasing the scale effect of the CE is also a challenge for the sectors and the platforms.

Finally, CE platforms and governments should strengthen the openness and sharing of data and introduce independent research institutions to assess the environmental impact scientifically. In addition, the public should be further encouraged to adopt green consumption ideas and adhere to moderate and rational consumption principles while fairly and objectively participating in the platform’s peer review process. In short, the government, platforms, and the public should work together to collaboratively govern the emerging CE and provide more safety and trust to society.

This paper provides a panorama of the initial sustainability assumptions and the current state of sustainability of the CE. Furthermore, it contributes to reviewing the current challenges by presenting a series of items adapted to the CE that can measure the level of sustainability of the CE and the CE platform itself. Thus, it not only has important theoretical significance for supplementing existing research in the field but has managerial implications for promoting the healthy and sustainable development of the CE and CE platforms. Although the conceptual framework and measurement items proposed in this study correspond to the entire CE industry, different sectors and platforms can still build suitable sustainability evaluation systems based on their operating models and the characteristics of the shared products or services. In addition, the results and findings can be used as the starting basis for decision-makers to identify problems and formulate more appropriate strategies and solutions to respond to the current challenges facing the CE in terms of sustainability.

Meanwhile, it can also help the public at large to better understand the potential of the CE in promoting economic, social, and environmental sustainability and the impact of their consumption criteria and behavior on CE sustainability. This may prompt users to re-examine their consumption motivations and patterns, thereby promoting consumers to establish sustainable consumption concepts and values. Furthermore, during the development process of the CE and CE platforms, developers should fully consider the overall impact on the sustainability of the economy, environment, and society instead of paying too much attention to just one aspect. Then, based on their actual conditions, platform companies can evaluate their sustainability according to the measurement items in this article and make targeted adjustments to their operating strategies and development directions to promote sustainable development better.

## Author Contributions

SS contributed to the conceptualization and first draft of the manuscript. ME contributed to supervision, project management, resources, editing, and revising the draft. Both authors contributed to the article and approved the submitted version.

## Conflict of Interest

The authors declare that the research was conducted in the absence of any commercial or financial relationships that could be construed as a potential conflict of interest.

## Publisher’s Note

All claims expressed in this article are solely those of the authors and do not necessarily represent those of their affiliated organizations, or those of the publisher, the editors and the reviewers. Any product that may be evaluated in this article, or claim that may be made by its manufacturer, is not guaranteed or endorsed by the publisher.
